# The potential value of crowdsourced surveillance systems in supplementing sentinel influenza networks: the case of France

**DOI:** 10.2807/1560-7917.ES.2018.23.25.1700337

**Published:** 2018-06-21

**Authors:** Caroline Guerrisi, Clément Turbelin, Cécile Souty, Chiara Poletto, Thierry Blanchon, Thomas Hanslik, Isabelle Bonmarin, Daniel Levy-Bruhl, Vittoria Colizza

**Affiliations:** 1 Sorbonne Université, INSERM, Institut Pierre Louis d’Epidémiologie et de Santé Publique (IPLESP), Paris, France; 2 UFR des sciences de la santé Simone-Veil, Université Versailles-Saint-Quentin-en-Yvelines, Versailles, France; 3 AP-HP, Service de Médecine Interne, Hôpital Ambroise Paré, Boulogne Billancourt, France; 4 Department of infectious diseases, Public Health France, Saint-Maurice, France

**Keywords:** influenza, surveillance, incidence, crowdsourced data, Internet, cohort

## Abstract

Participatory surveillance systems provide rich crowdsourced data, profiling individuals and their health status at a given time. We explored the usefulness of data from GrippeNet.fr, a participatory surveillance system, to estimate influenza-related illness incidence in France. **Methods:** GrippeNet.fr is an online cohort since 2012 averaging ca. 5,000 weekly participants reporting signs/symptoms suggestive of influenza. GrippeNet.fr has flexible criteria to define influenza-related illness. Different case definitions based on reported signs/symptoms and inclusions of criteria accounting for individuals’ reporting and participation were used to produce influenza-related illness incidence estimates, which were compared to those from sentinel networks. We focused on the 2012/13 and 2013/14 seasons when two sentinel networks, monitoring influenza-like-illness (ILI) and acute respiratory infections (ARI) existed in France. **Results**: GrippeNet.fr incidence estimates agreed well with official temporal trends, with a higher accuracy for ARI than ILI. The influenza epidemic peak was often anticipated by one week, despite irregular participation of individuals. The European Centre for Disease Prevention and Control ILI definition, commonly used by participatory surveillance in Europe, performed better in tracking ARI than ILI when applied to GrippeNet.fr data. **Conclusion**: Evaluation of the epidemic intensity from crowdsourced data requires epidemic and intensity threshold estimations from several consecutive seasons. The study provides a standardised analytical framework for crowdsourced surveillance showing high sensitivity in detecting influenza-related changes in the population. It contributes to improve the comparability of epidemics across seasons and with sentinel systems. In France, GrippeNet.fr may supplement the ILI sentinel network after ARI surveillance discontinuation in 2014.

## Introduction

Influenza surveillance systems aim to annually track influenza epidemics, detecting their start, monitoring their spatio-temporal spread, identifying populations at risk and circulating viruses, and estimating the impact on the community and healthcare structures [1]. Sentinel surveillance systems are based on primary care and report the weekly number of patients examined with influenza-related illness. Recently, a variety of non-traditional surveillance approaches have emerged [2-9] where large amounts of crowdsourced digital data produced by individuals enable such individuals to contribute to monitoring the health of their community and to provide authorities with additional characterisations of the epidemic. In the European Union, more than one third of the countries run a participatory surveillance system to monitor influenza epidemics, under the standardised framework of the Influenzanet network established in 2011 [2,10,11]. The Influenzanet network is a syndromic surveillance system based on voluntary self-reporting of symptoms by participants residing in countries, which are part of the Influenzanet.

Crowdsourced data bring novel issues regarding data analysis, due to their non-traditional nature. They refer to the dynamic participation of individuals having variable reporting behaviours along the season, individuals’ interpretation of the terms used for surveillance, and the correctness of their self-assessments. A small number of these aspects have been analysed in isolation in previous work [12-15], however a systematic evaluation is still missing. Crowdsourced surveillance is further complicated by the choice of the appropriate definition used to identify influenza cases. Because of the lack of specificity of influenza symptoms, national sentinel systems adopted influenza-like-illness (ILI) or acute respiratory illness (ARI) definitions, two of the most common quantitative indicators, however these indicators are defined at country level and no defined standard exists at the international level [16-18]. The evaluation and comparison of ILI and ARI quantitative indicators are critical for the sensitivity and specificity of resulting public health recommendations, though their performance in estimating the influenza-related illness epidemic characteristics non-trivially depends on age of the cases, circulating influenza subtypes, or medical practices [16,17]. Participatory systems have the advantage of being flexible in the case definitions to adopt, as case definitions can be built on different combinations of the symptoms collected, without requiring an a priori definition to be used by sentinel practitioners. Previous Influenzanet studies have mainly focused on a single case definition [14,15], thus it remains unclear how different combinations of self-reported symptoms perform in the accurate estimation of influenza incidences, which can be compared to available sentinel estimates.

Here we considered the case of France, where the participatory system GrippeNet.fr (GN) was established in 2012 as part of Influenzanet [19]. Previous work focused on individual-level epidemiological analyses, generally not possible in sentinel networks, allowed by the availability of individual data on demographic indicators, lifestyle, health variables, and attitudes [10,20,21]. In this study we focused on population-level indicators, and analysed crowdsourced incidence estimates comparing them to official estimates provided by sentinel systems. The 2012/13 and 2013/14 seasons were chosen for this work, because during this period two primary care surveillance systems monitoring independently ILI and ARI were available, against which we could assess the accuracy of GN analyses. 

We proposed and assessed different inclusion criteria accounting for individuals’ reporting and participation, and evaluated the accuracy of a set of case definitions. In addition, in light of the termination of the ARI sentinel surveillance system since 2014, we evaluated the possibility to use GN as a replacement for ARI surveillance. The overall aim was to: interpret GN weekly incidence estimates for influenza-related illness compared with available sentinel knowledge to provide continuous and robust data to monitor epidemic trends and intensities; propose a standardised approach to compute crowdsourced incidence, which could be extended to other Influenzanet countries allowing comparability across them and with national systems; learn from the only two seasons in France where three surveillance systems were in place (2 sentinel systems and 1 participative system) to assess GN’s potential benefits as an adjunct to the ILI sentinel influenza network in France.

## Methods

### GrippeNet.fr data collection

GN is a participatory surveillance system collecting voluntary reports of influenza-related symptoms through a dedicated website (https://www.grippenet.fr) where individuals also provide profile information [19]. Data are collected on a weekly basis through a symptoms survey [2,10,11]. Individuals are asked if they experienced any of the following 19 symptoms (or they can opt to report ‘no symptoms’): fever, chills, runny or blocked nose, sneezing, sore throat, cough, shortness of breath, headache, muscle/joint pain, chest pain, feeling tired or exhausted, loss of appetite, coloured sputum, watery/bloodshot eyes, nausea, vomiting, diarrhoea, stomach ache, other symptoms. If symptoms are reported, further questions are asked to characterise the participant’s behaviour (general practitioner (GP) consultation, drugs uptake) and to assess the syndrome (e.g. sudden onset, onset date). For reports of fever, a further question concerning the level of fever is asked, where the participant can input a measure of body temperature, however the response to this question is optional, so the temperature for fever is not always obtained. The full survey is provided as supplementary material (Supplement).

With respect to the general population, participants in GN are on average older and include a larger proportion of women [19]. GN population is however representative in terms of health indicators such as diabetes and asthma conditions.

### French surveillance networks

Two sentinel surveillance systems have been operating in France to monitor the influenza circulation in the country: Réseau Sentinelles (sentinel network, RS) estimating the weekly number of ILI cases [22]; GROG (Regional Influenza Surveillance Group) estimating the weekly number of ARI cases [23]. In 2014 GROG was discontinued and only ILI is currently monitored in France. Case definitions are reported in Table 1.

**Table 1:** Influenza-like-illness (ILI) and acute respiratory infection (ARI) case definitions used according to the surveillance systems, France, 2012/13 and 2013/14

Case definitions according to the surveillance system^a^		Sudden onset of symptoms	Fever^b^ and general symptoms	Respiratory signs
**ILI case definition**^a^				
Sentinel network (RS)		Yes	Fever ≥ 39 °C AND myalgia^c^	Yes
GrippeNet.fr^a^	ILI	Yes	Fever ≥ 39 °C AND pain^c^	Sore throat OR cough OR shortness of breath OR sneezing OR rhinorrhoea (runny or blocked nose)
	ILIf	Yes	Fever^b^ OR fever ≥ 39 °C AND pain^c^	Sore throat OR cough OR shortness of breath OR sneezing OR rhinorrhoea (runny or blocked nose)
	ILI–	Yes	Fever^b^ OR fever ≥ 38 °C AND pain^c^ OR headache	Sore throat OR cough OR shortness of breath
	ILI–f	Yes	Fever^b^ AND pain^c^ OR headache	Sore throat OR cough OR shortness of breath
**ARI case definition**^a^				
Regional Influenza Surveillance Group (GROG)		Yes	At least one sign suggesting an acute infection (fever or asthenia or headache or muscle pain, etc.)	Cough OR rhinitis OR coryza
GrippeNet.fr^a^	ARI	Yes	Fever^b^ OR chills OR malaise (feeling tired or exhausted) OR headache OR pain^c^	Cough OR rhinorrhoea OR sneezing
	ARI+	Yes	Fever^b^ OR chills OR malaise OR headache OR pain^c^	Cough OR rhinorrhoea OR sneezing OR sore throat OR shortness of breath OR coloured sputum/phlegm
	ECDC	Yes	Fever^b^ OR chills OR malaise OR headache OR pain^c^	Sore throat OR cough OR shortness of breath

ECDC: European Centre for Disease Prevention and Control.

^a^ Case definitions and inclusion criteria can vary in the participatory surveillance system GrippeNet.fr, so several possibilities are considered for this system.

^b^ Participants are free to report fever with or without measure of body temperature. In the table, the mention of ‘fever’ without a defining temperature threshold refers to self-reported fever without body temperature specification. Fever, without temperature specification, was considered in this study for the GrippeNet.fr case definitions because participatory surveillance systems are adopted in several countries in Europe and depending on individual preferences/habits may not always obtain the measures of body temperature.

^c^ For individuals above 5 years-old.

### Drug sales data

We considered sales data for 14 classes of medications in the EphMRA classification of interest for influenza [24]. Drug sales data are based on a representative sample of 14,000 pharmacies accounting for 60% of pharmacies in France.

### GrippeNet.fr data analyses: case definitions

Different case definitions can be built from GN symptoms (Table 1). We considered the GN_ILI_ and GN_ARI_ definitions most closely matching ILI and ARI definitions adopted by RS and GROG, respectively. We explored variations of GN_ILI_ by: relaxing the constraint on body temperature, whereby participants who declared a fever episode without specifying their body temperature were included in addition to participants reporting a body temperature ≥ 39 °C (GN_ILIf_); considering a lower temperature cut-off (≥ 38 °C), the inclusion of ‘headache’, and a restricted set of respiratory symptoms (GN_ILI_–); removing the constraint on body temperature (GN_ILI_–_f_). For the GN_ARI_ definition, we considered: a larger set of respiratory symptoms (GN_ARI +_ ) as well as the case definition of the European Centre for Disease Control and Prevention (GN_ECDC_), which is often used by Influenzanet studies [14,15]. Though formally an ILI definition, we included GN_ECDC_ in the ARI classification as it is less restrictive in the inclusion of ‘fever’ [25].

### GrippeNet.fr data analyses: incidence computation

We focused on the 2012/13 (from week 46 2012 to week 16 2013) and 2013/14 (from week 46 2013 to week 15 2014) influenza seasons. For each case definition and season, we estimated weekly incidence rates of cases from GN raw data considering the combination of several criteria (Table 2). The *raw* incidence time series is computed as the ratio between the number of GN participants declaring an episode in a given week and the total number of participants registered in the cohort from the beginning of the season until the week considered. 

**Table 2:** GrippeNet.fr (GN) incidence computation criteria, France

Computation criteria		Aim	Description
Raw criteria		Basic computation on raw data	All ILI/ARI episodes are considered. Weekly incidence rate is computed as the number of ILI/ARI episodes in the week divided by the total number of GN participants in the cohort from the beginning of the season up to that week
Episode reporting criteria	First survey exclusion	To account for first-time reporting bias	Weekly incidence rate is computed in the same way as for the raw criteria, but with the exclusion of the first report of each new participant
	Episode merging	To exclude reports on clinically unlikely different episodes	As raw, with the merging of consecutive ILI/ARI episodes within 2 weeks of each other
Participation criteria	Minimum number of symptoms reports (*m)*	To exclude individuals who have reported rarely	Denominator in the incidence computation is equal to the number of participants who filled ≥ *m* reports, *m = 2, 3*
	Participation window	To discard irregular participation and define a time window during which a participant can be considered actively engaged in the study	Denominator in the incidence computation is equal to the number of participants who reported at least once in the time window of *n* weeks before and after the reporting week; *n* = *0, 1, 2, 3, 4* (*n* = *0* means that participants are counted only in the week of reporting; *n* = *1* means that participants are counted in the week of reporting and also in the week before and in the week after)

ARI: acute respiratory infection; ILI: influenza-like-illness.

Subsequently, we first considered criteria accounting for different behaviours in reporting illness episodes. The *first survey exclusion* adjusts GN incidence rates by removing the result of the first survey of newly enrolled participants [5,13-15,26], as participants are more prone to report symptoms at their first report following enrolment [13,14]. To account for the lack of validation by a GP, the *episodes merging* criterion considers ILI/ARI episodes experienced within 2 weeks of previous ILI/ARI episodes to be part of the same illness episode [12,26]. Second, we considered inclusion criteria accounting for heterogeneous participation. We implemented a *minimum number m of symptoms reports* per individual throughout the season (*m* = 2 reports or *m* = 3 reports, including the first survey) to discard those with rare participation [13]. We considered the inclusion criterion of a *participation window* of *n* weeks around the reporting week (*n* = 0, 1, 2, 3, 4) to account for non-continuous participation. If *n* = 0 each participant is counted only in the week of reporting; if *n* = 1 each participant is considered to be part of the cohort in the week of reporting and also in the week before and the week after that, assuming e.g. that they forgot to connect online (analogously for *n* > 1). These criteria were combined in a stepwise progression (see results).

Incidence time series computed on these datasets were adjusted by age group (0–14, 15–44, 45–64, ≥ 65 years ) to account for the non-representative nature of the GN population [19], and smoothed through a linear filtering method [27] commonly used to filter out undesired spikes induced by large variations in enrolment (e.g. following a communication action). They were computed at the national and regional level (Ile-de-France, North-East, North-West, South-East and South-West, see Figure S1 in Supplement) with standard adjustment by region.

### Statistical analysis

GN incidence time series were compared with the official estimates of RS and GROG through the Pearson correlation coefficient (*r*) using the Bonferroni correction (p < 0.01) to evaluate the agreement of time series trends, and the normalised root mean square error (*e*, error divided by the average to compare measurements with different scales) to evaluate the agreement of influenza intensity. GN incidence trends were also compared with the drug sales time series through the correlation *r*.

### Ethics statement

GN was reviewed and approved by the French Advisory Committee for research on information treatment in the field of health (i.e. CCTIRS, authorisation 11.565), and by the French National Commission on Informatics and Liberty (i.e. CNIL, authorisation DR-2012–024).

## Results

A total of 6,046 individuals participated to GN submitting 77,875 symptom reports during the 2012/13 season (raw data, Table 3); 65% of them also participated in the 2013/14 season, which reached a total of 5,907 participants with 83,455 surveys filled in. The system had a mean weekly participation of 4,987 (± 1,224, standard deviation) and 5,112 (± 725) individuals in the two seasons. Participation criteria led to a progressive reduction of these numbers, stronger for the first season under study (25% reduction in 2012/13 vs 16% in 2013/14 with first survey exclusion, episodes merging, and *m* = 2 + *n* = 2 method compared with raw data).

**Table 3:** GrippeNet.fr seasons’ descriptive results, France, 2012/13 and 2013/14

Parameters	Criteria	2012/13	2013/14
		Week 46 2012–week 16 2013 (23 weeks)	Week 46 2013–week 15 2014 (22 weeks)
Number of participants	- Raw	6,046	5,907
Number of total symptoms survey	- Raw	77,875	83,455
Mean number of participants per week (± standard deviation)	- Raw	4,987 (± 1,224)	5,112 (± 725)
	- First survey excluded^a^ + episodes merged^b^ + *m* = 2	4,192 (± 1,039)	4,634 (± 665)
	- First survey excluded^a^ + episodes merged^b^+ *m* = 3	3,942 (± 914)	4,466 (± 605)
	- First survey excluded^a^ + episodes merged^b^ + *m* = 2 + *n* = 0	2,831 (± 574)	3,326 (± 353)
	- First survey excluded^a^ + episodes merged^b^ + *m* = 2 + *n* = 1	3,568 (± 718)	4,127 (± 452)
	- First survey excluded^a^ + episodes merged^b^ + *m* = 2 + *n* = 2	3,748 (± 775)	4,297 (± 489)
	- First survey excluded^a^ + episodes merged^b^ + *m* = 2 + *n* = 3	3,839 (± 814)	4,375 (± 512)
	- First survey excluded^a^ + episodes merged^b^ + *m* = 3 + *n* = 0	2,813 (± 575)	3,312 (± 357)
	- First survey excluded^a^ + episodes merged^b^ + *m* = 3 + *n* = 1	3,520 (± 709)	4,090 (± 455)
	- First survey excluded^a^ + episodes merged^b^ + *m* = 3 +* n* = 2	3,672 (± 756)	4,240 (± 487)
	- First survey excluded^a^ + episodes merged^b^ + *m* = 3 +* n* = 3	3,740 (± 785)	4,302 (± 506)

*m*: minimum number of reports of symptoms; *n*: number of participation weeks around the reporting week.

^a^ First report of each new participant is excluded.

^b^ Influenza-like illness/acute respiratory infections (ILI/ARI) episodes experienced within 2 weeks of previous ILI/ARI episodes are considered to belong to the same illness episode.

### GrippeNet.fr incidence computation methods

Overall, across all definitions of IRI and ARI respectively used based on GrippeNet.fr data, correlation was moderate (*r* = 0.57 ± 0.29) and strong (*r* = 0.69 ± 0.01) in the 2012/13 season for ILI and ARI, respectively, and very strong for both indicators in the 2013/14 season (*r* = 0.81 ± 0.03 for ILI, *r* = 0.86 ± 0.02 for ARI), when calculated on raw data (Table 4). Errors were much larger for ILI than for ARI comparisons (*e* = 3.4 ± 1.69 for ILI vs *e* = 0.8 ± 0.06 for ARI in 2012/13, and similarly for 2013/14 season). 

**Table 4:** Performance of computation methods in the comparison between GrippeNet.fr influenza-like illness and acute respiratory infection incidence estimates and official sentinel network estimates, France, 2012/13 and 2013/14

Criteria for participation and behaviour considered when analysing GrippeNet.fr data		2012/13		2013/14	
		Influenza-like illness	Acute respiratory infection	Influenza-like illness	Acute respiratory infection
Raw	*r*	0.57	0.69	0.81	0.86
	*e*	3.4	0.8	3.7	0.4
First survey excluded^a^	*r*	0.87	0.81	0.82	0.85
	*e*	1.0	0.2	2.2	0.2
First survey excluded^a^ + episodes merged^b^	*r*	0.84	0.81	0.80	0.84
	*e*	0.9	0.2	1.8	0.1
First survey excluded^a^ + episodes merged^b^ + *m* = 2	*r*	0.89	0.89	0.83	0.89
	*e*	1.0	0.2	2.0	0.1
First survey excluded^a^ + episodes merged^b^ + *m* = 3	*r*	0.88	0.90	0.82	0.89
	*e*	1.0	0.2	2.0	0.1
First survey excluded^a^ + episodes merged^b^ +* m* = 2 + *n* = 0	*r*	0.90	0.92	0.85	0.95
	*e*	1.7	0.7	3.4	0.7
First survey excluded^a^ + episodes merged^b^ +* m* = 2 + *n* = 1	*r*	0.90	0.92	0.85	0.93
	*e*	1.2	0.3	2.4	0.3
First survey excluded^a^ + episodes merged^b^ + *m* = 2 + *n* = 2	*r*	0.90	0.92	0.85	0.92
	*e*	1.1	0.2	2.2	0.2
First survey excluded^a^ + episodes merged^b^ + *m* = 2 + *n* = 3	*r*	0.90	0.92	0.85	0.91
	*e*	1.0	0.2	1.4	0.2
First survey excluded^a^ + episodes merged^b^ + *m* = 3 + *n* = 0	*r*	0.88	0.90	0.84	0.94
	*e*	1.7	0.7	3.2	0.6
First survey excluded^a^ + episodes merged^b^ + *m* = 3 + *n* = 1	*r*	0.89	0.91	0.84	0.92
	*e*	1.2	0.3	2.3	0.2
First survey excluded^a^ + episodes merged^b^ + *m* = 3 + *n* = 2	*r*	0.89	0.91	0.84	0.91
	*e*	1.1	0.2	2.1	0.2
First survey excluded^a^ + episodes merged^b^ + *m* = 3 + *n* = 3	*r*	0.88	0.91	0.84	0.91
	*e*	1.1	0.2	2.1	0.2

ARI: acute respiratory infection; *e*: mean error; ILI: influenza-like illness; *m*: minimum number of reports of symptoms; *n*: number of participation weeks around the reporting week; *r*: mean correlation.

In the table, incidence estimates based on the various ILI or ARI definitions from GrippeNet.fr were each used to compute respective correlations and errors against incidence estimates based on sentinel ILI and sentinel ARI. For each criteria for participation and behaviour in the table, a mean correlation and error across all GrippeNet.fr ILI definitions, and a mean correlation and error across all GrippeNet.fr ARI definitions are respectively presented.

^a^ First report of each new participant is excluded.

^b^ ILI/ARI episodes experienced within 2 weeks of previous ILI/ARI episodes are considered to belong to the same illness episode.

**Figure 1:**
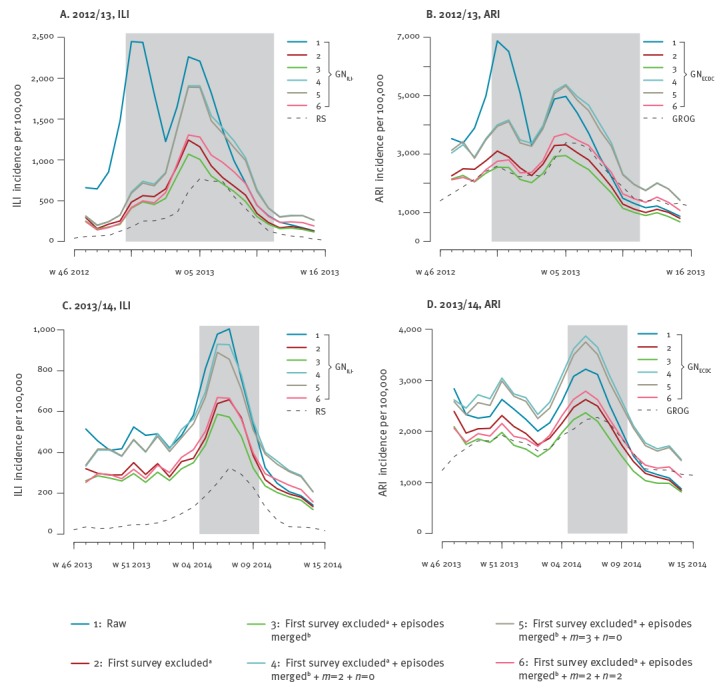
Comparison between official time series and GrippeNet.fr incidence curves using different computation methods, France 2012/13 and 2013/14

ARI: acute respiratory infection; GN: GrippeNet.fr; GROG: regional influenza surveillance group estimating the weekly number of ARI cases; ILI: influenza-like illness; RS: sentinel network estimating the weekly number of ILI cases; *m*: minimum number of reports of symptoms; *n*: number of participation weeks around the reporting week; w: week.

Excluding the first report of newly enrolled participants strongly increased correlation in the first season under study, whereas no effect was reported in the second season. Errors were considerably reduced in both seasons and for both indicators. This improvement is obtained as the *first survey exclusion* removes the large epidemic peak observed at the beginning of 2012/13 season that is not reported by sentinel sources, as illustrated in Figure 1 (panels A,B) for the particular examples of incidence estimates based on the GN_ILI_– and GN_ECDC _ILI definitions. Merging close consecutive episodes did not visibly affect incidence estimates. Adding inclusion criteria on participation of individuals slightly improved correlations, however strongly increasing the errors in the case *n* = 0 (Table 4), as it reduced the number of participants in the cohort (Table 3).

A to D show different computation methods for 2012/13 season (A, B) and 2013/14 season (C, D). A and C show GrippeNet.fr ILI (GN_ILI_–, coloured continuous lines) and RS (dashed black lines); B and D show GrippeNet.fr ARI (GN_ECDC_, coloured continuous lines) and GROG (dashed black lines). Shaded areas in all panels represent the 2012/13 and 2013/14 epidemic periods reported by RS.

^a^ First report of each new participant is excluded.

^b^ ILI/ARI episodes experienced within 2 weeks of previous ILI/ARI episodes are considered to belong to the same illness episode.

### Case definitions

To assess the accuracy of case definitions, we considered a baseline computation method composed of the episodes reporting criteria and participation criteria with *m* = 2 and *n* = 2. The largest correlation was obtained in both seasons with GN_ILI_– for ILI indicator with *r* = 0.95 (95% confidence interval (CI): 0.87–0.98) in 2012/13, and *r* = 0.91 (95%CI: 0.79–0.97) in 2013/14, Table 5) and with GN_ECDC_ for ARI indicator (*r* = 0.96; 95%CI: 0.89–0.98 in 2012/13, *r* = 0.93 (95%CI: 0.83–0.97) in 2013/14). Both case definitions brought an anticipation of the peak time of one week, with the exception of GN_ECDC_ in 2012/13 season. The overall smallest correlation was observed for GN_ILIf_ vs RS in both seasons.

**Table 5:** Performance of case definitions in the comparison between GrippeNet.fr incidence estimates and official estimates, France, 2012/13 and 2013/14

Comparison	ILI and ARI case definitions	2012/13			2013/14		
		Correlation *r* (95% CI)	Error *e*	Peak week difference^a^	Correlation *r* (95% CI)	Error *e*	Peak week difference^a^
GN ILI vs sentinel ILI	GN_ILI_ vs RS	0.89 (0.75–0.96)	0.62	2	0.84 (0.64–0.94)	0.51	0
	GN_ILIf_ vs RS	0.81 (0.58–0.92)	0.56	-1	0.83 (0.61–0.93)	1.40	-1
	GN_ILI_– vs RS	0.95 (0.87–0.98)	1.04	-1	0.91 (0.79–0.97)	2.35	-1
	GN_ILI_–_f_ vs RS	0.94 (0.86–0.98)	2.06	0	0.83 (0.62–0.93)	4.58	-1
GN ARI vs sentinel ARI	GN_ARI_ vs GROG	0.91 (0.79–0.96)	0.20	-1	0.93 (0.82–0.97)	0.18	-1
	GN_ARI +_ vs GROG	0.90 (0.78–0.96)	0.30	0	0.90 (0.76–0.96)	0.31	-1
	GN_ECDC_ vs GROG	0.96 (0.89–0.98)	0.13	0	0.93 (0.83–0.97)	0.16	-1
GN ILI vs sentinel ARI	GN_ILI_ vs GROG	0.77 (0.51–0.90)	0.95	2	0.84 (0.64–0.94)	0.94	0
	GN_ILIf_ vs GROG	0.82 (0.61–0.93)	0.87	-1	0.86 (0.68–0.94)	0.87	-1
	GN_ILI_– vs GROG	0.81 (0.59–0.92)	0.77	-1	0.86 (0.68–0.95)	0.80	-1
	GN_ILI_–_f_ vs GROG	0.88 (0.73–0.95)	0.63	0	0.89 (0.73–0.95)	0.67	-1
GN ARI vs sentinel ILI	GN_ARI_ vs RS	0.76 (0.49–0.90)	7.9	-1	0.66 (0.31–0.85)	17.5	-1
	GN_ARI +_ vs RS	0.76 (0.49–0.90)	8.7	0	0.60 (0.21–0.82)	19.7	-1
	GN_ECDC_ vs RS	0.85 (0.66–0.94)	7.4	0	0.66 (0.31–0.85)	17.0	-1

ARI: acute respiratory infection; CI: confidence interval; GN: GrippeNet.fr; GROG: regional influenza surveillance group estimating the weekly number of ARI cases; ILI: influenza-like illness; RS: sentinel network estimating the weekly number of ILI cases.

The different case definitions used in GN (GN_ILI_, GN_ILIf_, GN_ILI-, _GN_ILI-f_, GN_ARI_, GN_ARI +_, GN_ECDC_) are described in Table 1.

^a^ The difference is calculated as peak week of GN incidence minus peak week of official estimate.

**Figure 2:**
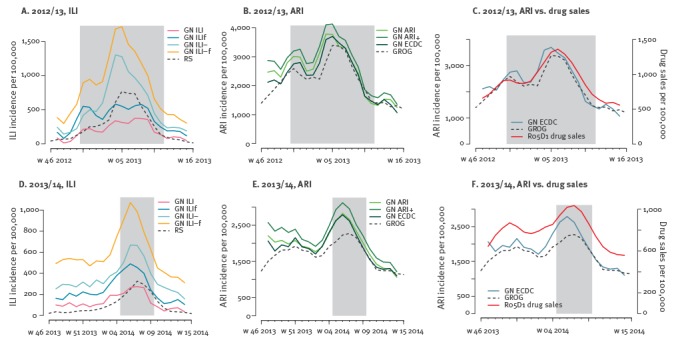
Comparison between official time series and GrippeNet.fr incidence curves using different case definitions within GrippeNet.fr, France 2012/13 and 2013/14

ARI: acute respiratory infection; ECDC: European Centre for Disease Prevention and Control; GN: GrippeNet.fr; GROG: regional influenza surveillance group estimating the weekly number of ARI cases; ILI: influenza-like illness; RS: sentinel network estimating the weekly number of ILI cases; w: week.

A higher agreement in the incidence trend and smaller errors were reported for ARI case definitions (*e* in the range 0.13–0.31) compared with ILI (0.51–4.58), with ARI curves rather close to each other and to GROG time series (Figure 2).

The different case definitions used in GN (GNILI, GNILIf, GNILI-, GNILI-f, GNARI, GNARI +, GNECDC) are described in Table 1.

A, B, D, E show different case definitions for the 2012/13 season (A, B) and the 2013/14 season (D, E). A and D show GrippeNet.fr ILI case definitions (coloured continuous lines) and RS (dashed black line); B and E show GrippeNet.fr ARI case definitions (coloured continuous lines) and GROG (dashed black line). C and F show GNECDC case definition, GROG, and drug sales. Shaded areas in all panels represent the 2012/13 and 2013/14 epidemic periods reported by RS.

The cross-comparison between ILI indicators from GN and ARI estimates provided by GROG led to strong correlations, slightly lower than those obtained between ILI indicators from GN and RS estimates (Table 5). ARI estimates from GN instead generally performed more poorly when compared with RS estimates (*r* = 0.85 vs 0.96 for GN_ECDC_ vs RS compared with GN_ECDC_ vs GROG in 2012/13; *r* = 0.66 vs 0.93 in 2013/14).

The GN_ILI_– and GN_ECDC_ case definitions for ILI and ARI, respectively, yielded the largest correlation with the plain antitussive drug class (R05D1, 0.89 ≤ *r* ≤ 0.92). The R05D1 sales trend reports a first peak early in the season, similarly to what estimated by GN (Figure 2; C,F).

### Regional incidence estimates

Correlations between GN regional estimates and corresponding official estimates are generally lower than the ones obtained at the national level, ranging from 0.61 in the North-West region to 0.87 in South-East in 2012/13, and from 0.64 in North-West, North-East, South-East to 0.75 in Ile-de-France in 2013/14 (Table S1 in Supplement). Weak non-significant associations were found between correlation coefficients and regional participations.

## Discussion

Despite the intrinsic differences between the self-selected population of GN and the medically assisted population considered by sentinel surveillances, epidemic trends from GN reports compare well with official sources. Notably, varying participation criteria led to uniformly very high correlations, suggesting that once adjusted for first-time reporting bias, crowdsourced indicators accurately summarise the seasonal epidemics, regardless of the regularity of participation of its volunteers. 

Epidemic intensities, on the other hand, are strongly dependent on participation criteria, as these alter the time-dependent size of the cohort. Higher intensities are expected in GN estimates compared with sentinel surveillance as the system also includes individuals not seeking medical care [20]. An appropriate frame of reference is currently missing because of the rather recent availability of this type of surveillance data, hindering the comparability of epidemic intensity across seasons and across surveillance systems. This still remains an unresolved challenge also in the framework of traditional sentinel networks in Europe, with countries reporting intensity levels based on national references, obtained with different methods, and depending on patterns of medical care consultation [28]. 

Given an arbitrary set of inclusion criteria (e.g. *m =* 2, *n* = 2) proposed here as a new standard to analyse crowdsourced data, an automated standardised method to define the epidemic threshold and a set of intensity thresholds should be put in place for the comprehensive analysis of crowdsourced data. For France, such implementation will soon be possible, after accumulating a long enough historical dataset from crowdsourced surveillance for thresholds estimation (minimum of six consecutive seasons for the moving epidemic method adopted in [28]). The advantage compared with traditional sources would be the rather seamless introduction of the method across all Influenzanet countries without additional burden on primary care surveillance, thus allowing the understanding of epidemic patterns across seasons and countries independently of health-seeking behaviour.

All case definitions performed rather well (*r* > 0.81) when comparing ILI case definitions on crowdsourced data with ILI sentinel surveillance, and analogously for ARI case definitions. Higher correlations were consistently obtained when less restrictive case definitions were considered. For ILI, lower temperature cutoffs or the addition of ‘headache’ in the general symptoms considerably improved the accuracy of GN case definition, even though headache is not a symptom which is associated with ILI by GPs in France. Previous work identified body temperature to be independently associated with virologically confirmed influenza with an increasing likelihood associated to rising temperature [16]. We found self-reported fever to be required as a mandatory criterion to accurately monitor ILI, in line with previous evidence [16,17], with best results obtained for body temperatures ≥ 38 °C. All case definitions having fever as an optional criterion better reproduce ARI incidence trends, confirming previous results on influenza cases [16,17]. Interestingly, comparisons with estimates from GROG, using a less restrictive case definition, were generally more accurate than with RS, adopting a more specific definition. 

All GN estimates reported the small peak of cases likely corresponding to the circulation of respiratory syncytial virus (RSV) viruses in the early weeks of the influenza epidemic period, visible only in ARI sentinel activity and identifiable by increased sales of antitussive drug class generally used for treatment. All these results seem to indicate that participants may find it easier to identify systemic symptoms rather than specific ones, given their lack of medical background. Interpretation of terms used in the case definitions may indeed be different between sentinel practitioners and the general population, and this may considerably impact the measure of influenza activity, as discussed in [18].

Our study shows that the ECDC definition, though formally an ILI definition, is a reliable definition for ARI but it performs rather poorly in tracking ILI in the general population. This finding is essential for participatory surveillance as the ECDC case definition is the one in use in Influenzanet. While a common case definition across countries is critical to standardise comparisons, different needs may emerge at the national level to monitor alternative indicators. Aguilera et al. [18] proposed sentinel networks to introduce a standardised definition to ensure comparability and compatibility of data, in addition to the domestic one historically adopted by each country. Based on self-reported symptoms that are digitally collected in real-time, participatory systems may easily introduce a double case definition, one for domestic use plus a standardised one, overcoming the challenges of increased workload of primary care surveillance.

The accuracy of GN in tracking ARI cases in the general population is specifically important in France, given that the ARI sentinel network was discontinued in 2014 due to changes in influenza surveillance practice. In this context, participatory surveillance may offer an alternative approach to continue the syndromic surveillance of ARI in the country, ensuring a high sensitivity in detecting changes in the epidemic throughout the season. In addition, GN could be coupled in the near future with virological confirmation from self-collected swabs from volunteering participants. With a pilot study within the United Kingdom system of Influenzanet [29], recent works confirmed indeed the feasibility and validity of self-collected swabs for respiratory virus surveillance [30,31]. The participatory approach would thus offer a novel solution to meet the recent recommendation by the World Health Organization prompting national sentinel networks to monitor also ARI episodes in order to describe a broader range of non-influenza viral pathogens [32]. This is particularly relevant in Europe where the large majority of countries (23/29) monitor exclusively ILI [28].

The flexibility of GN in establishing a case definition based on reported symptoms, without requiring the need for an a priori definition for GPs, is a critical difference with sentinel networks. Such flexibility could contribute to the identification and implementation of optimal case definitions per age class [16]. Most importantly, it would allow the adaptation of surveillance in real-time to monitor an unexpected or atypical clinical manifestation in the context of emerging influenza viruses. The system would also be able to offer continuous surveillance during a pandemic emergency when the public health infrastructure is expected to be overburdened.

An anticipation of 1 week in the incidence peak of GN vs sentinel network is found for the ILI case definition displaying the highest correlation, GN_ILI_–, as well as for the majority of case definitions explored. This suggests that self-reported digital data collected directly from the general population and analysed in a timely fashion can considerably reduce the time needed to produce sentinel estimates (that needs to account for the delay for consulting a primary care doctor and centralising sentinel data for analysis). Confirmed by other participatory systems [11,13-15,33], this feature may function as an important alert system for public health preparedness before reaching the highest weekly incidence value.

The study presents some limitations. GN population is not representative of the general population. While we were able to adjust on age and regional geographic distribution, further adjusting along additional indicators would strongly reduce the sample size in the stratification of the population, thus preventing significant analyses. The agreement found with GP incidence trends suggests however that these limitations have little effect once results are adjusted on basic indicators.

The small number of participants at the regional level led to noisier GN estimates. Correlations with official sources were found to be weakly associated with GN participation in the region, though non-significant. It would be interesting to increase the sample size and systematically assess such dependency on the European scale to identify the minimum number of participants per geographical area to obtain reliable results.

The study was conducted on two seasons only as this is the only time period during which two independent sentinel networks existed in France to monitor different indicators of influenza-related illness against which GN surveillance data could be evaluated.

## Conclusion

GN is an online participatory system providing flexibility and a richness of data offering several opportunities to track and analyse epidemics due to pathogens causing influenza-suggestive symptoms. We evaluated the accuracy of seven influenza case definitions and of different inclusion criteria accounting for variable reporting and participating behaviours of individuals in the online cohort. GN estimates are in very good agreement with the official trends, proving the system to be rather sensitive in detecting influenza-related changes in the population and often anticipating the peak, regardless of the regularity of participation. Evaluation of influenza intensity will benefit from the estimation of epidemic and intensity thresholds for crowdsourced surveillance data once enough seasons will become available. Less restrictive case definitions could be chosen for surveillance purposes as they are found to be more accurate. The ECDC definition for ILI currently adopted by the European participatory system Influenzanet would perform poorly in tracking ILI epidemics, therefore we suggest Influenzanet countries to adopt additionally a case definition including fever and body temperature with a low cutoff (≥ 38 °C) as compulsory criteria. The standardised method we proposed is essential for the comparability and compatibility of crowdsourced estimates allowing the understanding of epidemic patterns across seasons and countries. In France, GN would represent the ideal candidate to supplement the current sentinel network by monitoring ARI after the termination of GROG and prospectively including self-swabbing.
